# The buffering effect of social capital for daily mental stress in an unequal society: a lesson from Seoul

**DOI:** 10.1186/s12939-023-01875-w

**Published:** 2023-04-10

**Authors:** Sungik Kang, Joo-Lim Lee, Ja-Hoon Koo

**Affiliations:** 1 Department of Urban and Regional Development, Hanyang University, Seoul, South Korea; 2 URI Urban Institute Co., Ltd, Seoul, South Korea; 3 Department of Urban and Regional Development, Hanyang University, 222 Wangsimni-Ro, Seongdong-Gu, Seoul, 04763 South Korea

**Keywords:** Daily mental stress, Mental health, Social capital, Economic inequality, Gini coefficient

## Abstract

This study attempted to illustrate whether mental health deterioration could be alleviated by high social capital in an environment with high economic inequality. Daily mental stress was employed as a mental health factor when analyzing the association with economic inequality in the Seoul Survey data. Regarding social capital, community trust and altruism were included as cognitive dimensions, and participation and cooperation were included as structural dimensions in each model. The first finding showed a significantly positive relationship between economic inequality and daily stress, meaning that, like other mental health problems, daily mental stress is also high in regions with high economic inequality. Second, the slope of the daily stress increased in respondents with high social trust and participation was alleviated in an economically unequal environment. This indicates that social trust and participation have a buffering effect by moderating the slope of daily stress in societies with high inequality. Third, the buffering effect differs depending on the social capital factor. The buffering effect of trust and participation showed in an unequal environment, while the buffering effect of cooperation showed regardless of the unequal environment. In summary, social capital factors showed the effect of relieving daily mental stress in the relationship with economic inequality. Also, the buffering effect of social capital on mental health may show different aspects for each element.

## Background

Many people suffer from mental illnesses due to problems such as depression, anxiety, and stress. In 2017, approximately 100 million people worldwide had mental disorders, and the number of years lived with disabilities related to mental illness has been on the rise [1]. Moreover, illnesses such as depression have become common in modern times [2, 3], and depression symptoms are highly prevalent worldwide, in high- as well as low-to-middle-income countries [4]. As suicide [5, 6] and homicide rates [7] related to mental disorders such as depression and emotional anxiety incur substantial social costs, we need to understand various factors that stimulate mental disorders.

Along with mental health problems, increasing socioeconomic inequality is also a prominent social issue. Thus, various studies have been conducted on increases in mental health problems and socioeconomic inequality [8–12]. In an unequal environment, psychosocial health has theoretically developed into absolute income and health, relative income and health, and income inequality and health [13]. Wilkinson [14], who presented contextual theory, explained that income inequality, like air pollution, inevitably leads to deterioration of physical and mental health, even for relatively wealthy people. An unequal society is not only associated with poor physical health [15], but also intensifies competition on the social ladder, resulting in poor mental health [16].

A society with high socioeconomic inequality is an environment in which the disparity in status between classes widens, and social issues and mental disorders related to dominance and subordination can occur more frequently [12]. Furthermore, social status becomes more critical as society becomes more unequal and anxiety regarding social status tends to increase [17]. In other words, societies and countries with high socioeconomic inequality are more likely to be exposed to mental disorders such as anxiety, deprivation, and frustration because the competition for social status intensifies [18]. This also explains why violence, family disruption, crime, and homicide related to mental illness frequently occur in a socioeconomically unequal society where the importance of social status is further emphasized [14, 19].

The main empirical results for socioeconomic inequality and mental health are as follows. Pickett and Wilkinson [16] analyzed 12 major developed countries, such as the United States, Europe, and Japan, and showed a high correlation between the level of national inequality and the rate of mental disorders, such as mood disorders, anxiety, and impulse control. Layte and Whelan [17] investigated 31 European countries and showed that the more unequal societies there are, the higher the insecurity status. Pabayo et al. [11] analyzed the state-level income inequality and depression in the United States, showing that significant depression among women was high in unequal states. Furthermore, Pabayo et al. [20], in examining post-traumatic stress disorder (PTSD), did not associate inequality with persistent or recurrent PTSD. In contrast, inequality was significantly associated with incident PTSD (OR 1.30; 95% CI, 1.04, 1.63). Chiavegatto Filho et al.’s [21] study in the São Paulo metropolitan area found that depression was higher in unequal areas (OR 1.76, 95% CI 1.21, 2.55), although it was not associated with anxiety.

Social capital is a critical psychosocial factor related to public health [22], and various studies have recently analyzed the relationship between social capital and mental health [23–25]. Social capital, generated by trust, reciprocity, and mutual aid, refers to mutual relationships with the community in an institutionalized society and the resources that could be obtained through this network [26–28]. Berkman and Krishna [29] explained that individuals’ social networks, participation, and support are linked not only to health habits, but also to psychological health, such as depression and emotional regulation. The more we belong to social networks and the more support we have, the better is our mental health and the lower the incidence of cancer, cardiovascular disease, and stroke [29–31]. Almedom and Glandon [32], who analyzed social capital and mental health, explained that the psychological mechanism of belonging to a neighborhood and community is beneficial to mental and emotional health for both adolescents and adults.

Social capital consists of cognitive and structural dimensions [33]. The cognitive dimension is social capital expressed through perceptions, thoughts, and attitudes, while the structural dimension is social capital formed through network formation and actual behavior [34]. It is important to separately explore the mechanisms that affect health according to social capital’s cognitive and structural dimensions [24, 34–36]. The cognitive dimension of social capital tends to affect mental health through psychosocial pathways such as self-esteem, social support, and social buffers for stressful life incidents [14, 37, 38]. Factors such as social support, altruism, and a culture of mature respect can enhance psychological health [39]. In contrast, structural social capital can benefit mental health in the form of instrumental resources, sharing valuable health knowledge, and providing information support to local facilities [40, 41]. Putnam [42] explained that the higher the social network participation, the better is the physical and psychological health status through social safety nets, such as health information and nursing.

Economic inequality is correlated not only with mental health but also with social capital. Neighborhoods with high economic inequality tend to have low social capital. The mechanism of economic inequality and social capital is that the greater the socioeconomic gap, the greater the possibility that social distance between classes increases and, as a result, social exchanges between neighbors decrease [43]. According to Putnam [42], the twentieth century in the United States was a period in which society progressed with increasing economic inequality damaging social capital. Residential segregation along the hierarchy of income inequality leads to the social segregation of communities and negatively affects the formation of social capital [44]. Consequently, societies with large economic disparities tend to have increased social stratification and competition for status, resulting in decreased social capital [45].

Various studies have analyzed the hypothesis that increasing economic inequality reduces social capital for communities [46–49]. For example, Kawachi et al. [46], who studied economic inequality and social capital in 39 U.S. states, found that higher income inequality was associated with lower group belonging and social capital. Using the U.S. General Social Survey, Gold et al. [47] illustrated through path analysis that income inequality is negatively related to social trust. Putnam [42], using the 50 U.S. states, found that the more unequal the income distribution in a state, the lower the level of social capital in that state. Several studies show that regions with greater inequality have lower levels of individual social capital as well as group social capital. Eric's [48] study using the World Values Survey confirmed that, like individual income levels, regional income inequality is a variable that plays an important role in explaining social trust. In China, Dai et al. [49] indicated that not only income inequality but also wealth inequality had a significant association with low social trust.

The above studies have highlighted economic inequality as a factor associated with mental health and social capital as a major factor influencing mental health. At the same time, several studies have emphasized the interaction between economic inequality and social capital. In other words, these variables tend to influence each other simultaneously. Therefore, social capital should be a key consideration in exploring the relationship between economic inequality and mental health. However, since related studies that consider social capital as important are limited, we explored the moderating effect of social capital on the relationship between economic inequality and mental health. Similar studies include mortality [46], violence [50], and happiness [51] in the relationship between inequality and social capital. Kawachi et al. [46] showed that social capital mediates the relationship between income inequality and mortality. Kennedy et al. [50] demonstrated that income inequality and violent crime rates involving firearms are mediated by local social capital. Delhey and Dragolov [51] highlighted that trust in local residents indirectly affects the relationship between economic inequality and happiness. However, there is an understanding gap in whether social capital significantly alleviates on unstable mental health in unequal environments.

In this vein, we attempted to illustrate that the threat to mental health in an economically unequal society can be mitigated by social capital. In other words, this study aimed to demonstrate the moderating effect of social capital on the relationship between economic inequality and mental health. In addition, evidence of severe mental health, such as depression and traumatic stress, has been provided for economic inequality and mental health research. However, knowledge of everyday mental stress is lacking. As mental stress experienced in daily life has different characteristics from depression, anxiety, and suicide, this study focused on daily mental stress. Furthermore, daily stress is the basis of cardiovascular disease, cancer, anxiety, and depression [52–54]. As it is important in determining the psychological quality of life [25], daily mental stress must be explored as a target. In addition, most studies on economic inequality, social capital, and mental health have been conducted in Western countries. However, since inequality and mental health issues have been on the rise in Asian countries, this study analyzed the citizens of Seoul, in Asia.

The assumptions of this study are as follows: (1) The first assumption is that the level of daily mental stress in regions with high economic inequality is high. Existing studies have demonstrated that depression, anxiety, and traumatic stress are associated with intense mental health in highly unequal societies [9, 11, 16, 20]. The first assumption is developed that neighborhoods with higher levels of economic inequality might be associated not only with higher levels of mental health but also with higher levels of daily stress. (2) The second assumption is that, in regions with high economic inequality, high daily mental stress levels are alleviated by social capital. Economic inequality has been correlated to mental health [8–12] and social capital [46–49]. In addition, as social capital plays a significant positive role in mental health [23–25], the second assumption is developed that social capital would have a positive effect on the positive relationship between economic inequality and mental health. (3) The third assumption is that the buffering role of social capital differs according to the characteristics of its cognitive and structural dimensions of social capital. Several researchers have emphasized that cognitive and structural types of social capital have different effects on mental health and need to be explored separately by the types [37, 38, 40, 41]. The third assumption is developed that the moderating effect of social capital on the relationship between economic inequality and mental health might differ by type of social capital.

## Methods

### Study site

Seoul, the capital of the Republic of Korea, is a large city with a population of approximately 10 million. Each autonomous district (Gu) in Seoul has different socioeconomic characteristics and lifestyles depending on its function and role [55].The Gus are divided by natural barriers, such as rivers, mountains, and topography, and large-scale infrastructure, such as highways [56]. Gangnam-gu, Seocho-gu, and Songpa-gu are residential areas with large-scale apartment complexes, whereas Dobong-gu, Nowon-gu, Gangbuk-gu, and Seongbuk-gu are traditional residential areas in Seoul. Jongno-gu and Jung-gu are residential areas filled with Seoul’s history and long-standing traditional culture, and Yongsan-gu is an area where various foreigners live in dense clusters. The knowledge-based industry is concentrated in Guro-gu, and the digital media industry has developed in Mapo-gu. In summary, each Gu in Seoul has different socioeconomic functions and living standards.

### Data and variables

This study measures the economic inequality index for each autonomous district using housing price data from the Ministry of Land, Infrastructure, and Transport (MOLIT). Although Seoul and South Korea do not disclose regional inequality indices, the MOLIT provides housing prices as measurable data on the degree of economic inequality. According to the Bank of Korea in 2015, about 75% of people’s assets were housing assets, so the disparity in individual housing transaction prices would represent the level of economic inequality in the region [57]. As the MOLIT discloses actual housing price transaction data, such as price and location in all regions annually, users can identify individual housing prices and price disparities in the autonomous district. The housing price transaction volume from 2015 to 2019 (the study period) was as high as 126,000 cases (2019) and as low as 192,000 cases (2015).

This study measures the degree of regional economic inequality using the Gini coefficient, which is a commonly used index among the indicators to measure the degree of inequality [58, 59]. The index shows a value between 0, indicating an equal society, and 1, indicating an unequal society. A society with a Gini coefficient close to 0 means a society with small economic disparity among its members, and a society with a Gini coefficient close to 1 means a society with large economic disparity among its members. Although the Gini coefficient was designed to measure income disparity, the index is also used to measure disparity and distribution in diverse fields [60] in housing price distribution [9, 61], happiness inequality [62], and bacterial aggregation [63]. Based on the basic principle of the Gini coefficient, this study calculated the regional economic inequality index by accumulating housing prices from the lowest to the highest by replacing income.

The economic inequality index formula based on the regional housing price is as follows (Eq. 1). The formula for the Gini coefficient G for any individual housing price *i* and *j* is as follows: where* n* is the number of individual housing prices in the area, $\mu$ is the average housing price in a region, ${x}_{i}$ is the price of housing *i*, and ${x}_{j}$ is the price of housing *j*. The housing price Gini coefficient was calculated by comparing the prices of all housing in pairs and dividing the difference $|{x}_{i}-{x}_{j}|$ by the average housing price $\mu$. In other words, the housing price Gini coefficient, an index of economic inequality, is close to 1 in regions with large housing price disparities and close to 0 in regions with small housing price disparities.

1 $${G}_{n}=\frac{1}{2{\mu n}^{2}}\sum_{i=1}^{n}\sum_{j=1}^{n}|{x}_{i}-{x}_{j}|$$

This study used Seoul Survey data for social capital and individual socioeconomic variables. The Seoul Survey data have been investigated annually since 2003 for the Seoul Metropolitan Government to establish urban policy plans and to understand citizens’ lifestyles scientifically. The primary questions of the Seoul Survey are regarding the behavioral patterns and perceptions of citizens regarding social issues. Every year, the data are selected using stratified cluster sampling and surveyed through face-to-face interviews. The sample comprises about 20,000 households or 40,000 people, each year, and includes household heads and members aged ≥ 15 residing in Seoul [64]. In particular, the Seoul Survey is suitable for analyzing social capital as it examines various social capital variables, such as community trust, community participation, and altruism [65].

This study classified social capital into cognitive and structural variables using Seoul Survey data. The cognitive dimension is related to thinking and perception, while the structural dimension is related to networks and behavior [33, 66]. This study employed trust in a regional society and altruism for vulnerable people as the cognitive dimension. Moreover, we used community participation and cooperative networks with neighbors as structural dimensions. As trust is based on relationships and networks and enhances the utility of social resources, it is an alternative form of social capital [67]. Putnam [42] explained that altruism toward society is an important factor in diagnosing social capital, because people with good social networks spend time and money on charity and volunteer work for the community. Furthermore, Putnam [42, 68] argued that the degree of participation indicates the social capital level in society, as participation promotes and enhances collective norms and trust. Flap [69] specifies that social capital is defined as the number of neighbors who can give and receive help, intensity of help, and availability of resources in the group. All social capital variables in the data were investigated for 5 years (2015–2019).

As a cognitive dimension variable, the question about trust in regional society was “Do you trust your local neighbors, family, and public institutions?” The question about altruism for vulnerable people was “Do you think social consideration is necessary for the disabled, the elderly, and women?” The question about community participation as a structural variable was, “Do you usually participate in community activities such as local meetings, volunteer work, and civic groups?” Finally, the question about cooperative networks with neighbors was, “Do you have neighbors who give and receive help?” Participants answered yes or no to all these questions. As a result of the reliability analysis of social capital factors, Cronbach’s alpha was 0.280 and the correlation coefficient was < 0.4, which showed low consistency as a social capital factor. Thus, we empirically analyzed each social capital factor.

Each analysis model included a set of socioeconomic control variables that were applied to mental health and stress studies. Based on Han [24], who analyzed perceived stress and social capital, the control variables of this study were gender (1 = female and 0 = male), age (10–19, 20–29, 30–39, 40–49, 50–59, and 60 + years), educational attainment (1 = college graduate or above and 0 = below), employment status (1 = employed and 0 = unemployed), marital status (1 = married and 0 = unmarried), monthly income (continuous variable based on 1,000 Won), and home ownership (1 = ownership and 0 = rented house). Based on He et al. [70], who studied relative income disparity and mental health, this study included household size (continuous variable based on the number of family members) and self-rated health status (0 = very poor, 10 = very good). Based on Chen and Koenig’s [71] study, which analyzed traumatic stress, religion (1 = yes and 0 = no) was included as a control variable. Finally, based on Schiffrin and Nelson’s [72] study on stress and happiness, we included satisfaction with economic status (0 = very low, 10 = very high) and satisfaction with social life (0 = very low, 10 = very high). Table 1 shows the descriptive statistics for all control variables.

**Table 1:** Statistic summary of variables

Variables (description)	Obs	Mean	Std. Dev	Min	Max
Dependent variable					
Daily mental stress (1 = yes, 0 = no)	96,160	0.718	0.450	0	1
Independent variables Economic inequality variable					
Housing price gini coefficient index	125	0.204	0.039	0.106	0.323
Social capital variable					
Trust in regional society (1 = yes, 0 = no)	96,160	0.725	0.447	0	1
Altruism for vulnerable people (1 = yes, 0 = no)	96,160	0.627	0.484	0	1
Community participation (1 = yes, 0 = no)	96,160	0.720	0.449	0	1
Cooperative networks with neighbors (1 = yes, 0 = no)	96,160	0.758	0.428	0	1
Socioeconomic covariate					
Gender (1 = female, 0 = male)	96,160	0.481	0.500	0	1
Age (1 = teenager/2 = 20 s/3 = 30 s/4 = 40 s, 5 = 50 s, 6 = over 60)	96,160	3.984	1.539	1	6
Education (1 = college graduate or above, 0 = below)	96,160	0.323	0.468	0	1
Total monthly income (1,000 Won^a^)	96,160	4,871	2,134	250	9,250
Household size (number of family members)	96,160	3.081	1.035	1	5
Home ownership (1 = yes, 0 = no)	96,160	0.592	0.492	0	1
Marital status (1 = yes, 0 = no)	96,160	0.662	0.473	0	1
Employed (1 = yes, 0 = no)	96,160	0.850	0.357	0	1
Religious beliefs (1 = yes, 0 = no)	96,160	0.457	0.498	0	1
Self-rated health status	96,160	6.518	2.203	0	10
Satisfaction with economic status	96,160	6.318	1.513	0	10
Satisfaction with social life	96,160	7.059	1.401	0	10

*Obs* Observation, *Std. Dev*. Standard deviation

^a^1,000 Won is roughly equivalent to USD 0.8$ (2022 exchange rate)

### Main effect test

This study employed binary multilevel logit analysis as the analysis method. As the housing price inequality index is a regional variable for individual mental stress, a multilevel analysis methodology was employed. The inequality index was analyzed using models corrected for both fixed and random effects. We interpreted a model with a high fit between the two models. Model suitability was judged by the AIC and BIC values, and the model with low AIC and BIC values ​​was selected as suitable. Finally, since the Seoul Survey data are pooled multi-year survey results, we corrected the model with a time-fixed effect to compensate for year-specific heterogeneity based on Kang et al. [65] and Kim and Jin [73].

For the autonomous districts (*j*) and time (*t*) to which each respondent (*i*) belongs, the formula for daily mental stress is given below. ${Y}_{ijt}$ is the mental stress value and is considered the dependent variable of individual respondent *i* in autonomous districts *j* and time *t*. ${G}_{jt}$ is the housing price inequality index vector, and ${X}_{ijt}$ is the individual’s socioeconomic characteristic vector. The first model analyzes ${u}_{j}$ as a regional fixed effect (Eq. 2). $\sum {\lambda }_{j}{D}_{j}$ represents the regional fixed effect, and ${D}_{j}$ is a dummy variable that takes the value of 1 if it corresponds to the *j*-th region and 0 otherwise. The second model considers ${u}_{j}$ a regional random variable and analyzes it as a multilevel model (Eq. 3). For all models, $\sum {\eta }_{t}{S}_{t}$ represents the time-fixed effect, and ${S}_{t}$ is a dummy variable that takes a value of 1 if it corresponds to the *t*-th time and 0 otherwise:

2 $$\mathrm{g}\left({Y}_{ijt}\right)=log\frac{\mathrm{Pr}({Y}_{ijt})}{1-\mathrm{Pr}({Y}_{ijt})}=\alpha +{\beta G}_{jt}+\beta {X}_{ijt}+{u}_{j}+{u}_{t}+{e}_{ijt},$$

$${u}_{j}=\sum_{j=1}^{n-1}{\lambda }_{j}{D}_{j}, {u}_{t}=\sum_{t=1}^{T-1}{\eta }_{t}{S}_{t}$$

3 $$\mathrm{g}\left({Y}_{ijt}\right)=log\frac{\mathrm{Pr}({Y}_{ijt})}{1-\mathrm{Pr}({Y}_{ijt})}=\alpha +{\beta G}_{jt}+\beta {X}_{ijt}+{u}_{j}+{u}_{t}+{e}_{ijt}$$

$${u}_{t}=\sum_{t=1}^{T-1}{\eta }_{t}{S}_{t}$$

### Moderating effect test

This study analyzes the function of social capital as a moderating effect in the relationship between economic inequality and daily stress. Although MacKinnon [74] and Preacher and Hayes [75] emphasize the causal relationship between independent variables and mediators in the mediation effect method, the relationship between economic inequality and social capital is ambiguous based on the social capital components. For example, Lyu et al. [76] found that social trust and networks are lower in neighborhoods with greater economic inequality, but the effect is not statistically significant for social participation. As such, because the relationship between economic inequality and social capital tends to vary depending on the factors, this study analyzes daily mental stress according to economic inequality and social capital using moderation effects rather than mediation effects. For the moderating effect, the interaction variable of the predictor and moderator is added to the model, and the effect of the moderating variable is verified with statistical significance [77]. In this study, the moderating effect was verified by adding the interaction term ${G}_{jt}{S}_{ijt}$, which is a combination of economic inequality and social capital, to the model, as shown in Eq. 4.

4 $$\mathrm{g}\left({Y}_{ijt}\right)=log\frac{\mathrm{Pr}({Y}_{ijt})}{1-\mathrm{Pr}({Y}_{ijt})}=\alpha +{\beta G}_{jt}+{\beta S}_{ijt}+{\beta G}_{jt}{S}_{ijt}+\beta {X}_{ijt}+{u}_{j}+{u}_{t}+{e}_{ijt}$$

$${u}_{t}=\sum_{t=1}^{T-1}{\eta }_{t}{S}_{t}$$

## Results

### Effects of the housing price inequality on daily mental stress

The multilevel random-effects model was interpreted as a random-effects model because it was more suitable. Table 2 presents the results of the analysis of daily stress according to the degree of housing price inequality index and social capital. Model 1 applies the housing price inequality index, Model 2 applies economic inequality and social capital, Model 3 applies only social capital, and Model 4 includes all the covariates. Models 1, 2, and 4 show a negative relationship between the economic inequality index and daily mental stress. In Model 4, the housing price inequality index (OR = 1.459; 95% CI = 1.222, 1.742) and daily mental stress are negatively related; that is, the results confirm the hypothesis that daily stress is high in areas with high economic inequality.

**Table 2:** Analysis results for association between economic inequality, social capital, and daily mental stress

Perceived daily stress	Model 1	Model 2	Model 3	Model 4
	OR (95% CI)	OR (95% CI)	OR (95% CI)	OR (95% CI)
Constant	1.490 (0.810 1.604)	0.718 (0.505 1.021)	2.002 (1.807 2.219))	2.398 (1.346 4.271)
Economic inequality variable				
Housing price gini coefficient index	1.712 (1.436 2.041)	1.746 (1.461 2.085)		1.459 (1.222 1.742)
Social capital variables				
Trust in regional society		1.369 (1.305 1.436)	1.367 (1.303 1.434)	1.396 (1.328 1.468)
Altruism for vulnerable people		1.430 (1.385 1.477)	1.430 (1.384 1.476)	1.483 (1.434 1.534)
Community participation		1.193 (1.151 1.237)	1.192 (1.150 1.236)	1.274 (1.226 1.325)
Cooperative networks with neighbors		0.728 (0.703 0.754)	0.728 (0.703 0.754)	0.731 (0.705 0.758)
Covariates				
Gender				1.103 (1.070 1.136)
Age				0.839 (0.828 0.850)
Education				1.049 (1.014 1.085)
ln(Income)				1.099 (1.064 1.134)
Household size				1.033 (1.015 1.051)
Home ownership				0.957 (0.927 0.988)
Marital status				0.994 (0.956 1.033)
Employed				1.194 (1.141 1.249)
Religious beliefs				1.002 (0.972 1.033)
Self-rated health				0.698 (0.691 0.705)
Satisfaction with economic status				0.975 (0.964 0.987)
Satisfaction with social life				1.091 (1.077 1.106)
N	96,160	96,160	96,160	96,160
Log likelihood	-56,110.6	-55,556.8	-55,578.4	-51,713.3
AIC	112,235.4	111,135.8	111,176.8	103,472.8
BIC	112,301.7	111,240.0	111,271.6	103,690.7

### Effects of social capital on daily mental stress

Models 3 and 4 in Table 2 provide the results on the relationship between social capital factors and daily mental stress. In Model 4, all the social capital variables showed a statistically significant relationship with daily stress. Among social capital variables, trust (OR = 1.396, 95% CI = 1.328, 1.468), altruism (OR = 1.483, 95% CI = 1.433, 1.534), and participation (OR = 1.274, 95% CI = 1.226, 1.325) were positively associated with daily mental stress. That is, the higher the level of trust, altruism, and participation, the higher the level of daily mental stress. In contrast, cooperation (O.R. = 0.731, 95% CI = 0.705, 0.758) and daily stress showed a negative relationship; that is, respondents who had a high level of a cooperative network of neighbors had low daily stress.

### Moderating effects of social capital on stress in association with the housing price inequality

**Fig. 1:**
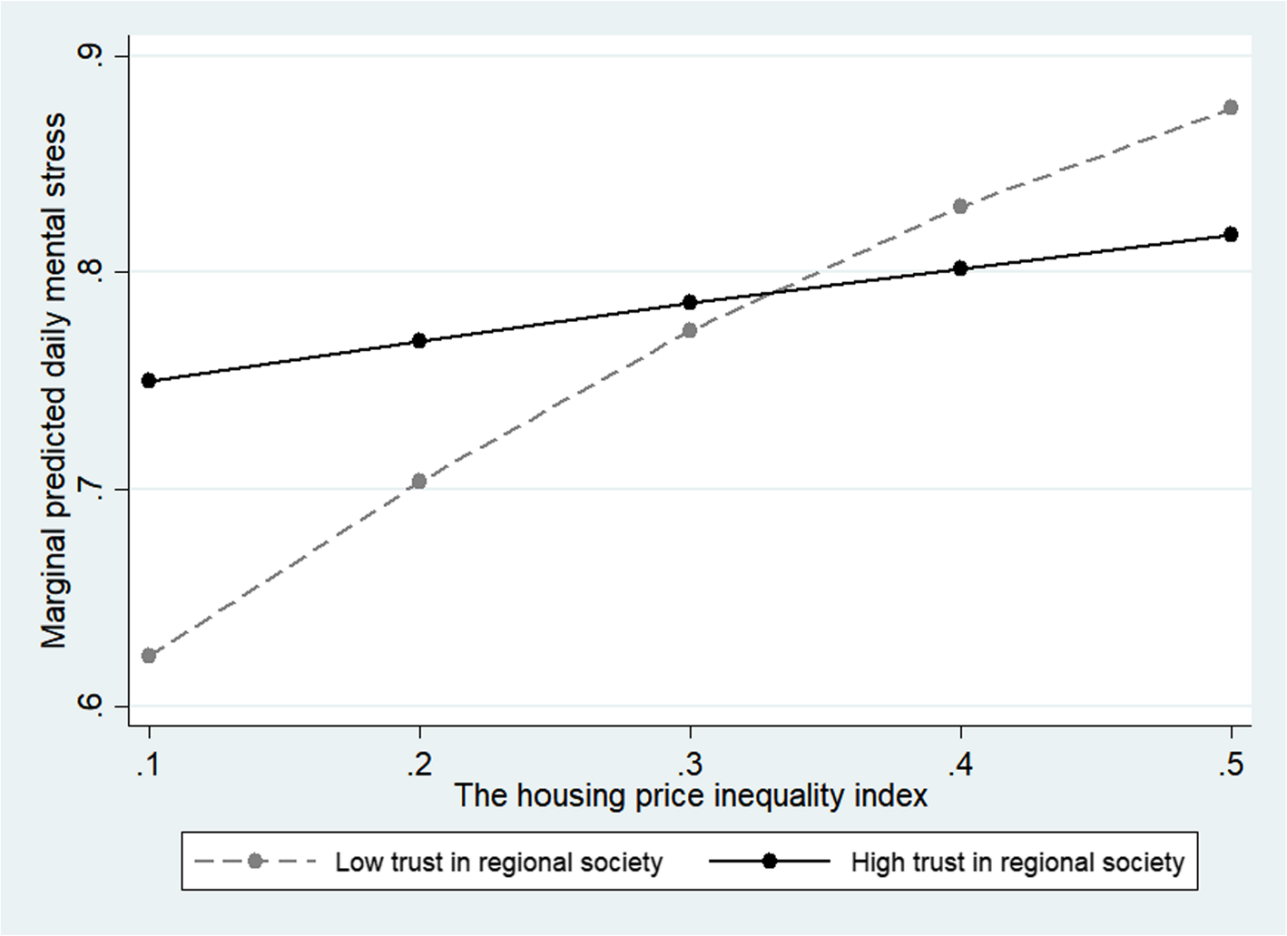
Moderate effect of trust level for regional society

**Fig. 2:**
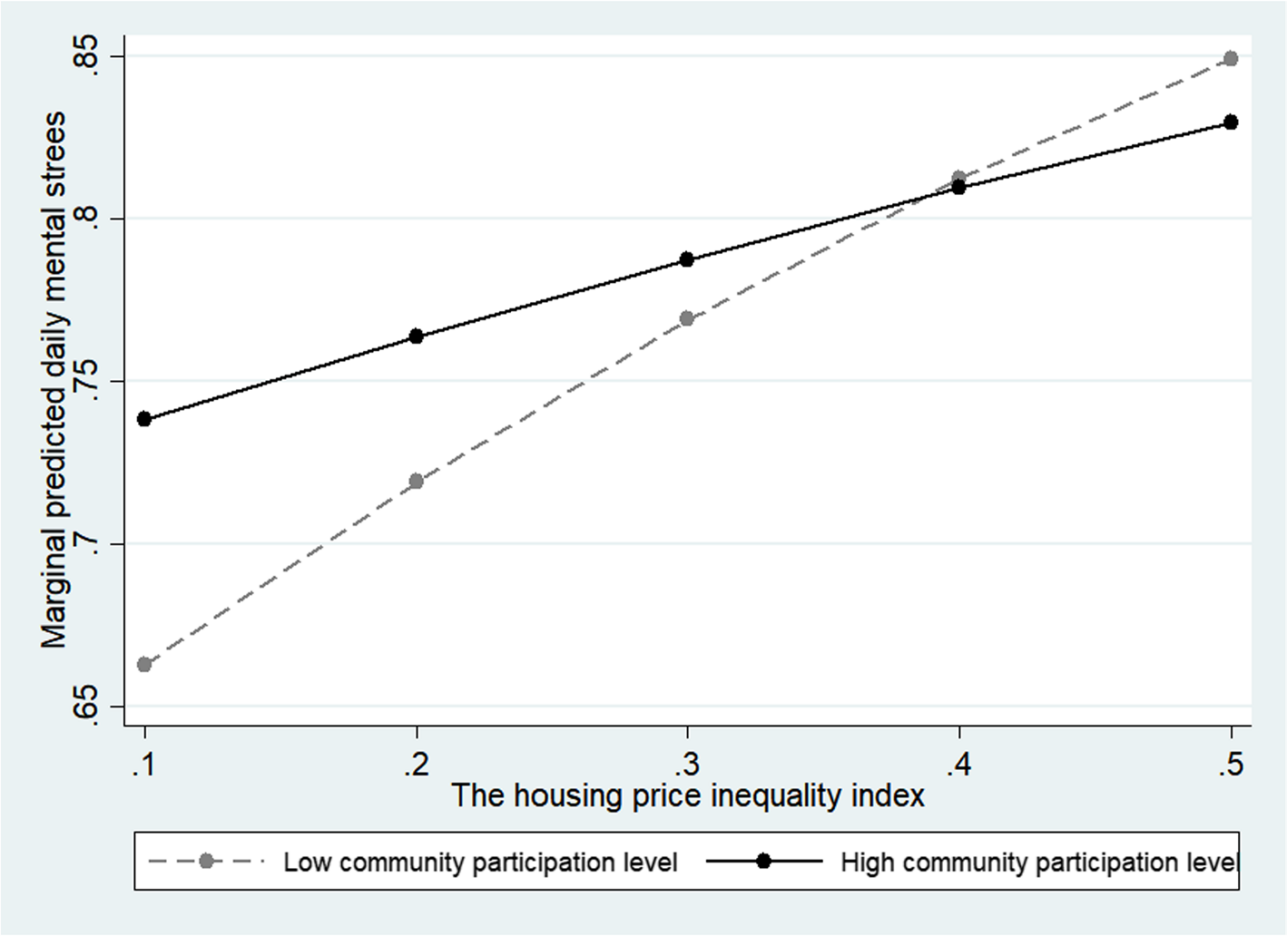
Moderate effect of community participation level

**Fig. 3:**
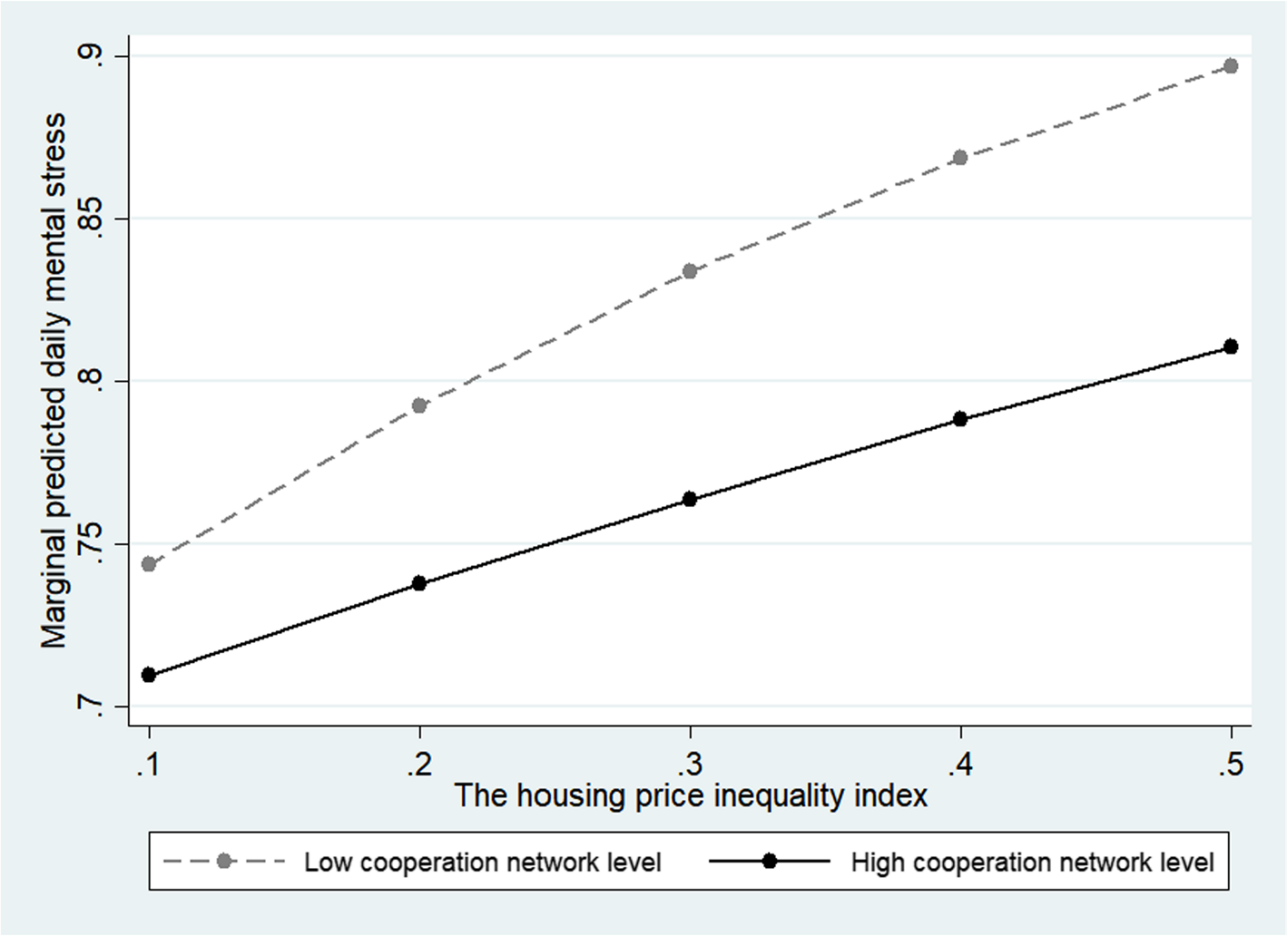
Moderate effect of cooperation network level

The multilevel random-effects model was interpreted as a random-effects model because it was more suitable. Models 5 and 6 in Table 3 include the moderating effects of the housing price inequality index on the cognitive and structural factors of social capital, respectively, and Model 7 includes all moderating effects. Model 7 shows a moderating effect on the interaction variables of trust, participation, and cooperation among social capital factors. The trust, participation, and cooperation all showed the effect of relieving daily stress in the relationship with the housing price inequality index. In the case of trust (OR = 0.807; 95% CI = 0.730–0.892) and participation (OR = 0.902; 95% CI = 0.823–0.988), the increase in stress was moderate in high-inequality environments compared to those with lower levels (Figs. 1 and 2). In the case of cooperation (OR = 0.869, 95% CI = 0.790 0.957), the daily mental stress increase was moderate as the housing price inequality increased in the high-level respondents compared to the low-level respondents (Fig. 3). In other words, we confirmed the hypothesis that social capital factors have different buffering effects on the relationship between the housing price inequality index and daily mental stress.

**Table 3:** Social capital moderate effect with economic inequality for daily mental stress

Perceived daily stress	Model 5	Model 6	Model 7
	OR (95% CI)	OR (95% CI)	OR (95% CI)
Constant	1.585 (0.869 2.893)	1.712 (0.936 3.132)	1.124 (0.600 2.104)
Economic inequality variable			
Housing price gini coefficient index	1.814 (1.488 2.212)	1.705 (1.402 2.073)	2.132 (1.719 2.644)
Social capital variables			
Trust in regional society	2.107 (1.713 2.592)	1.400 (1.331 1.472)	2.152 (1.748 2.648)
Altruism for vulnerable people	1.726 (1.443 2.065)	1.488 (1.438 1.539)	1.700 (1.421 2.035)
Community participation	1.272 (1.224 1.323)	1.497 (1.247 1.798)	1.557 (1.296 1.870)
Cooperative networks with neighbors	0.736 (0.710 0.763)	0.991 (0.816 1.204)	0.974 (0.801 1.184)
Covariates			
Gender	1.103 (1.071 1.137)	1.103 (1.070 1.136)	1.103 (1.071 1.137)
Age	0.839 (0.828 0.850)	0.839 (0.828 0.850)	0.839 (0.828 0.850)
Education	1.048 (1.012 1.084)	1.049 (1.013 1.085)	1.047 (1.012 1.084)
ln(Income)	1.096 (1.062 1.132)	1.100 (1.065 1.135)	1.097 (1.063 1.133)
Household size	1.034 (1.017 1.052)	1.033 (1.015 1.051)	1.034 (1.017 1.052)
Home ownership	0.957 (0.927 0.988)	0.956 (0.926 0.988)	0.956 (0.926 0.987)
Marital status	0.993 (0.955 1.032)	0.993 (0.955 1.032)	0.992 (0.954 1.032)
Employed	1.194 (1.141 1.249)	1.193 (1.140 1.248)	1.193 (1.140 1.247)
Religious beliefs	1.002 (0.972 1.033)	1.002 (0.972 1.033)	1.002 (0.972 1.033)
Self-rated health	0.698 (0.691 0.705)	0.698 (0.691 0.705)	0.698 (0.691 0.705)
Satisfaction with economic status	0.975 (0.964 0.987)	0.975 (0.964 0.987)	0.975 (0.964 0.987)
Satisfaction with social life	1.092 (1.078 1.106)	1.092 (1.078 1.106)	1.092 (1.078 1.106)
Interactions			
Trust*Inequality index	0.814 (0.737 0.900)		0.807 (0.730 0.892)
Altruism*Inequality index	0.926 (0.847 1.011)		0.934 (0.855 1.020)
Participation*Inequality index		0.921 (0.841 1.009)	0.902 (0.823 0.988)
Cooperation*Inequality index		0.859 (0.781 0.945)	0.869 (0.790 0.957)
N	96,160	96,160	96,160
Log likelihood	-51,700.3	-51,706.7	-51,693.6
AIC	103,450.7	103,463.6	103,441.2
BIC	103,687.6	103,700.4	103,697.0

## Discussion and conclusions

The results of this study suggest that an unequal environment is related not only to severe mental disorders such as traumatic stress, suicidal ideation, and depression, but also to the stress commonly felt in daily life. As per Durkheim [78] and Wilkinson’s [79, 80] hypothesis, economic inequality is related to mental disorders, and previous studies have empirically analyzed inequality with traumatic stress [20], status anxiety [17], depression [11, 21], and suicidal ideation [9]. Although the mental health problems in previous studies are close to severe mental illness, the daily stress in this study is a mild mental health problem felt in daily life. This study demonstrates that mild mental health problems are associated with unequal environments. In other words, the economically unequal environment is related not only to a high degree of psychological trauma, but also to daily psychological stress. The results of this study highlight that a society with high inequality negatively influences the mind through psychological mechanisms such as competition for status, relative deprivation, and unequal rewards [14, 79, 81], which are also related to perceived stress in daily life. In other words, this suggests that daily stress increases as inequality increases.

As a result of the moderating effect of social capital, the findings show that social capital had a buffering effect on daily mental stress in an economically unequal society. Our results suggest that social capital’s role in mental health increases with social disparity and an unequal environment. This finding sheds light on the fact that when trust in the public and local communities is low, stress can increase because of distrust in government agencies and local communities in problem-solving about widening gaps when economic inequality increases. This implication supports the finding that low trust in the federal government and financial institutions was the basis for the Occupy Wall Street protests [82]. In this vein, in a society where inequality grows, people with high trust in the government and community look forward to solutions at home. However, people who lack trust are likely to experience stress on the street.

Regarding the buffering effect of community participation, the psychological stress of respondents with a low level of community participation increased more steeply than that of those with a high level of community participation when inequality increased. This finding suggests that community participation plays a positive role in mental health [83–86] in the context of high economic inequality. Regarding the buffering effect of the cooperative network among neighbors, it was found that when for the respondents with a low cooperative network level, the daily stress level rose more steeply than for the respondents with a high cooperative network level, when economic inequality increased. This result indicates that people with good cooperative networks have greater psychological stability in a society with disrupted social balance and high competition for status, such as socioeconomic inequality.

The next discussion point is the ambivalence of social capital, which can be positive or negative, depending on the social situation. The analysis of social capital on mental health showed that people with a highly cooperative network had low daily mental stress. Having a network in which neighbors can cooperate means a social safety net for giving and receiving help in difficult times, and this safety net lowers mental stress. This finding supports previous studies that have demonstrated positive effects of social capital on mental health [32, 36, 41].

In contrast, trust in regional society, altruism toward vulnerable people, and community participation showed opposite results from cooperative networks with neighbors. Our findings show that people with high trust in regional society, altruism toward vulnerable people, and community participation had high daily stress. These findings show the double-sidedness of social capital pointed out by Portes [87]. Several critics have argued that the social assets generated by social networks are not always acceptable to the community [88] or its members [89]. Sometimes, coerced social capital might be harmful [90], and several studies have described that high social capital leads to high levels of psychological distress [91] and poor psychological health [92, 93]. As high social capital carries the burden of considering and caring for other people’s problems, it has not only a positive aspect, but also a great burden and liability [29]. In sum, similar to the results of Weil et al. [93], this finding indicates that it is necessary to recognize that social capital may be positive or negative for mental stress, depending on the characteristics of each social capital element and socioeconomic situation. For example, in community participation, high community participants had high daily mental stress in an equal society. In contrast, high community participants had low daily mental stress in an unequal society.

This study illuminates the relationship between economic inequality that can be visually recognized, and daily mental stress. Since it is difficult to determine individual income unless bank account information is disclosed, it is difficult for people to recognize income inequality visually. However, it is possible to estimate the regional economic inequality index through the housing price gap because people can judge housing value based on the characteristics such as the size of a house, appearance, location, public and private service amenities, and transportation infrastructure. Therefore, we conducted our analysis based on the hypothesis that people could perceive the degree of inequality through local housing and that this perceived inequality would be related to mental health. For example, Roseto residents were aware of this and did not reveal a social status gap outwardly; thus, they could maintain a high social interaction and health status [94], suggesting that the visually perceived level of inequality is low if residents live in a community that does not show off, even in a society with a large disparity. Thus, the community can escape negative consequences such as mental health deterioration or decreased social capital.

This study has several limitations. First, it assumes a negative impact of economic inequality on mental health. However, similar to many previous studies [29], it was not possible to distinguish whether the negative effects of economic inequality on mental health were mediated through physical health or had a direct effect on mental health. Second, this study analyzed the buffering effect of social capital on mental health through social capital factors, such as trust, altruism, participation, and cooperation. However, other factors, such as kinship, emotional support, and belonging, represent social capital [33]. In addition, as the results suggest, social capital factors have different characteristics. Therefore, further studies are needed to consider the various types of social capital. Finally, it is important to analyze social capital by dividing it into bonding-type social capital, a dimension of homogeneous social exchange, and bridging-type social capital, a dimension of heterogeneous social exchange [39, 95]. Although bonding- and bridging-type social capital have different characteristics, it was not possible to analyze these types separately.

## Data Availability

All data and material is available through the website of the Seoul Survey Data Portal (https://kossda.snu.ac.kr/handle/20.500.12236/15540).
